# Influenza Virus and Glycemic Variability in Diabetes: A Killer Combination?

**DOI:** 10.3389/fmicb.2017.00861

**Published:** 2017-05-22

**Authors:** Katina D. Hulme, Linda A. Gallo, Kirsty R. Short

**Affiliations:** ^1^School of Biomedical Sciences, The University of Queensland, BrisbaneQLD, Australia; ^2^Mater Research Institute, The University of Queensland, BrisbaneQLD, Australia; ^3^Australian Infectious Diseases Research Centre, The University of Queensland, BrisbaneQLD, Australia

**Keywords:** influenza, diabetes, glucose, hyperglycemia, glycemic oscillations

## Abstract

Following the 2009 H1N1 influenza virus pandemic, numerous studies identified the striking link between diabetes mellitus and influenza disease severity. Typically, influenza virus is a self-limiting infection but in individuals who have a pre-existing chronic illness, such as diabetes mellitus, severe influenza can develop. Here, we discuss the latest clinical and experimental evidence for the role of diabetes in predisposing the host to severe influenza. We explore the possible mechanisms that underlie this synergy and highlight the, as yet, unexplored role that blood glucose oscillations may play in disease development. Diabetes is one of the world’s fastest growing chronic diseases and influenza virus represents a constant and pervasive threat to human health. It is therefore imperative that we understand how diabetes increases influenza severity in order to mitigate the burden of future influenza epidemics and pandemics.

## Introduction

Every year, approximately 5–15% of the world’s population are infected with influenza virus (Shirey et al., 2013). Of the three types of influenza virus (A, B, and C), influenza A virus is the most common cause of respiratory illnesses in humans. Influenza A virus typically causes an acute and self-limiting infection characterized by symptoms such as myalgia, fever, and a dry cough. However, in patients with one or more underlying medical conditions, influenza A virus can cause severe, and even fatal, disease (Short et al., 2015). Influenza thus represents a significant healthcare challenge for the 21st century, where the majority of people have more than one medical ailment (Vos et al., 2015). This interaction between chronic disease and influenza was particularly evident after the 2009 H1N1 influenza pandemic (Kumar et al., 2009). Specifically, this pandemic highlighted that people with diabetes suffered from more severe influenza than people with no underlying medical condition (Allard et al., 2010; Wilking et al., 2010). Here, we review the currently available literature on the role of diabetes in the pathogenesis of influenza virus. We further highlight the specific roles that high, and/or oscillating, blood glucose levels may play in the severity of influenza virus.

## Diabetes Mellitus and its Vascular Complications

Diabetes mellitus affects 415 million people worldwide and this figure is projected to increase to 642 million by the year 2040 (International Diabetes Federation, 2015). Diabetes is characterized by chronic hyperglycemia and is classified into two main types. Type 1 diabetes accounts for approximately 10% of all cases and is most commonly caused by an autoimmune condition affecting insulin production. In contrast, type 2 diabetes accounts for approximately 85–90% of cases and is characterized by insulin resistance and compromised insulin secretory capacity. All individuals with diabetes have an increased risk of developing a number of serious complications. Diabetes is a leading cause of blindness, limb amputations, end-stage kidney failure, and cardiovascular disease (The Emerging Risk Factors Collaboration, 2011). Chronic hyperglycemia is thought to underlie the development of complications and, as such, a primary aim in the management of diabetes is to improve blood glucose control (Gallo et al., 2015). Blood glucose control is typically assessed by measuring a patient’s HbA1c. The term HbA1c refers to glycated hemoglobin, i.e., when hemoglobin joins with glucose and becomes ‘glycated.’ The average lifespan of red blood cells is approximately 2–3 months, meaning that HbA1c provides an estimate of long-term blood glucose control. Interestingly, however, there is no guarantee that achieving near-normal HbA1c levels (HbA1c < 7%), particularly in patients with long-standing type 2 diabetes, will prevent the onset and progression of vascular complications. Using traditional therapies, such as metformin, sulphonylureas and insulin, near-normal HbA1c levels were achieved in numerous trials but mortality from cardiovascular disease was either not affected (The U.K. Prospective Diabetes Study Group, 1998; The Advance Collaboration group, 2008; Duckworth et al., 2009) or increased (The Action to Control Cardiovascular Risk in Diabetes Study Group et al., 2008). In the latter study, the trial was prematurely stopped due to a significant increase in short-term mortality following intensive glucose lowering (The Action to Control Cardiovascular Risk in Diabetes Study Group et al., 2008). The lack of a definitive conclusion in this field has dampened clinical urgency to normalize HbA1c levels and suggests that other factors may contribute to the development of diabetic complications.

## Influenza Virus in Diabetes Mellitus

Prior to the 2009 H1N1 pandemic, several studies had already suggested that diabetes enhanced the severity of influenza (Diepersloot et al., 1987; Valdez et al., 1999). Valdez et al. (1999) showed that from 1986 to 1989, people with diabetes were more likely to have pneumonia and influenza recorded on their death certificate than people without diabetes. However, the most extensive body of evidence regarding this relationship emerged following the first influenza pandemic of the 21st century: the 2009 H1N1 pandemic. Numerous clinical studies suggested that people with diabetes were a key susceptibility group for severe H1N1 infections (see **Table 1**). For example, in Canada, diabetes tripled the risk of hospitalization after infection with the 2009 H1N1 virus and quadrupled the risk of admission to the intensive care unit (Allard et al., 2010). Similarly, in Germany, diabetes doubled the risk of a fatal outcome after infection with the 2009 virus (Wilking et al., 2010). Whilst the majority of clinical studies suggest a role for diabetes in increasing influenza severity, this synergism was not observed in all studies (see **Table 1**). Taken together, these data suggest that the relationship between diabetes and influenza may vary depending on the diabetic patient population in question. It is also important to note that many patients with diabetes have various other conditions that can increase (or decrease) the severity of influenza. For example, approximately 90% of patients living with type 2 diabetes are overweight, and obesity is an independent risk factor for severe influenza (Morgan et al., 2010). Adjusting clinical analyses for the presence of these other illnesses has yielded contradictory results (see **Table 1**), highlighting the need for further research in this area. Nevertheless, consistent with the majority of clinical observations, murine models demonstrate that diabetes increases susceptibility to severe infections with both seasonal and highly pathogenic influenza virus strains (see **Table 2**).

**Table 1:** The role of diabetes in the severity of 2009 pandemic influenza.

Year/s observed	Country	Sample size (% diabetes^1^)	Diabetes type^2^	Primary outcome(s) measured	Results^3^	Findings/conclusions	Reference
April 2009 – June	USA^4^	668 (2%)	n/a	Hospitalization	ND: 311/658 (47%), D: 5/10 (50%)	Diabetes did not affect the rate of	Garcia et al., 2015
2010				ICU admission	ND: 114/658 (17%), D: 2/10 (20%)	hospitalization or ICU admission	
1 January – 1 December 2009	Spain	11,499 (9%)	97 Type I 936 Type II	Death	ND: 244/10,416 (2%), D: 38/1,033 (4%)	No difference in fatality risk in any age group between those with and without diabetes	Jiménez-García et al., 2013
16 April – 30 August 2009	Canada	716 (7%)	n/a	Hospitalization ICU admission Death	ND: 283/666 (43%), D: 38/50 (76%) ND: 39/666 (6%), D: 8/50 (16%) ND: 12/666 (2%), D: 3/50 (6%)	Diabetes was associated with an increased risk of hospitalization.	Gilca et al., 2011
26 April – 26	Canada	1,479 (9%)	n/a	Non-severe outcome	ND: 1,089/1,342 (81%), D: 82/137 (60%)	The risk of a severe outcome was	Campbell et al., 2010
September 2009				Non-fatal ICU admission Death	ND: 194/1,342 (15%), D: 42/137 (31%) ND: 59/1,342 (4%), D: 13/137 (10%)	greatest among patients with diabetes	
1 May – 31 December 2009	USA	66 (32%)	n/a	ICU admission	ND : 19/45 (42%), D: 10/21 (48%)	No difference in pre-existing comorbidities between ICU and non-ICU patients	Venkata et al., 2010
25 May – 1 July 2009	Canada	162 (13%)	9 Type I 13 Type II	ICU admission	ND: 21/140 (15%), D: 10/22 (45%)	Diabetes tripled the risk of hospitalization and quadrupled the risk of ICU admission once hospitalized	Allard et al., 2010
12 June – 5 December 2009	Spain	304 (50%)	n/a	ICU admission Death	ND: 69/252 (27%), D: 97/252 (38%) ND: 15/252 (6%), D: 30/252 (12%)	Worse outcomes in patients with diabetes may be a consequence of the higher prevalence of underlying medical conditions rather than diabetes itself	Cortes et al., 2011
24 July 2009 – 3 March 2010	French West Indies and French Guiana	241 (3%)	n/a	Severe infection Death	ND: 27/234 (12%), D: 3/7 (43%) ND: 30/234 (13%), D: 0/7 (0%)	Diabetes was associated with a higher risk of severe influenza	Barrau et al., 2012
1 October – 23 December 2009	China	155 (13%)	n/a	Death	ND: 20/128 (16%), D: 7/20 (35%)	Diabetes is likely to be an additional risk factor compared to obesity alone	Xi et al., 2010
November 2009 – January 2010	France	1,266 (10%)	n/a	ICU admission Death	Diabetes was a risk factor for ICU admission ND: 241/1139 (21%), D: 34/127 (27%)	Diabetes is likely to be an additional risk factor compared to obesity alone for ICU admission but not death	Hanslik et al., 2010
March – December 2010	Brazil	4,740 (2%)	n/a	Hospitalization	ND: 1,948/4,632 (42%), D: 72/108 (67%)	Diabetes was identified as a risk factor for hospitalization	Lenzi et al., 2012

*^1^Percentage rounded to nearest whole number.*

*^2^n/a: Diabetes type was not specified.*

*^3^D: Patients with diabetes; ND: Patients without diabetes.*

*^4^Patients aged 18 years or younger.*

**Table 2:** Animal models of diabetes and influenza.

Mouse model	Diabetes type modeled	Influenza A virus subtype	Measure of disease severity	Findings	Reference
STZ-induced diabetes in BALB/c mice	I	H1N1	Lung viral titers Lethal dose 50	Diabetic mice had increased influenza virus titers and a lower lethal dose 50 compared to non-diabetic mice.	Zhu et al., 2005
STZ-induced diabetes in BALB/c mice	I	H5N1	Lung viral titers Lethal dose 50	Diabetic mice had increased influenza virus titers, a lower lethal dose 50 and a more persistent viral infection compared to non-diabetic mice.	Wu et al., 2010
RIP-K^b^ transgenic diabetic mice	I	H3N2	Lung viral titers Weight loss	There was a significant correlation between blood glucose levels and influenza virus titers in diabetic mice. Diabetic mice had increased influenza virus titers but no difference in weight loss compared to non-diabetic mice.	Reading et al., 1998
BKS.Cg-+*Lepr*db/+*Lepr*db/Jcl (diabetic mice)	II	H1N1	Lethal dose 50 Death	Diabetic mice had a lower lethal dose 50 and a higher mortality rate compared to non-diabetic mice.	Ito et al., 2015

## A Role for Hyperglycemia

At present, the mechanisms by which diabetes can increase the severity of influenza remain unclear. There is a growing body of evidence that hyperglycemia can increase the incidence and severity of bacterial infections. For example, diabetic patients with an HbA1c level > 7% had a three times increased risk of active tuberculosis compared to those with an HbA1c level < 7% (Leung et al., 2008). Similarly, diabetic patients with hyperglycemia (>7% HbA1c) were more likely to develop *Klebsiella pneumoniae* liver abscess than diabetic patients with controlled glycaemia (Lin et al., 2013). It has also been reported that individuals with diabetes are more likely to suffer from infection-related mortality following a kidney allograft than non-diabetic patients (Hayer et al., 2014). However, in a recent systematic review of a range of surgical specialities, the relationship between preoperative HbA1c levels and postoperative complications, including mortality from infection, was less convincing (Rollins et al., 2016). The authors noted that their retrospective analyses contained heterogenous datasets of small sample sizes and that further research, specifically dedicated to addressing this topic, is warranted (Rollins et al., 2016).

With regards to respiratory tract infections, Rayfield et al. (1982) noted a striking positive correlation with the mean plasma glucose levels in patients with diabetes. These findings may reflect, in part, the immunosuppressive effects of hyperglycemia. Hyperglycemia can reduce neutrophil degranulation (Stegenga et al., 2008), impair complement activation (Ilyas et al., 2011) and impair phagocytosis (Alexiewicz et al., 1995) – all of which can increase the severity of bacterial infections as well as viral infections such as influenza. However, to date, there have been only limited experimental studies directly addressing the role of hyperglycemia in the pathogenesis of influenza virus.

Elevated blood glucose levels can directly increase glucose concentrations in airway secretions (Philips et al., 2003). *In vitro* exposure of pulmonary epithelial cells to elevated glucose concentrations significantly increased influenza virus infection and replication (Kohio and Adamson, 2013), suggesting that hyperglycemia may increase viral replication *in vivo*. Elevated glucose levels may also serve to suppress the anti-viral immune response (Reading et al., 1998). In a mouse model of type I diabetes, susceptibility to influenza was associated with a reduction in the anti-viral activity of collectins (Reading et al., 1998). This immunosuppression was driven by hyperglycemia as disease susceptibility could be reversed with insulin treatment (Reading et al., 1998). These findings are consistent with studies of patients infected with highly pathogenic avian influenza, whereby hyperglycemia was associated with a fatal outcome (Wiwanitkit, 2008). Interestingly, in a mouse model of type II diabetes, susceptibility to influenza was associated with an impairment in the recruitment of macrophages to the lung (Ito et al., 2015). However, whether this phenotype was driven by hyperglycemia or other physiological changes associated with diabetes remains to be determined. Hyperglycemia may also affect pulmonary function such that influenza virus-induced respiratory dysfunction is exacerbated in patients with diabetes. In animal models of disease, diabetes is associated with numerous structural changes to the lung including augmented permeability of the vasculature and a collapsed alveolar epithelium (Popov and Simionescu, 1997). In patients with diabetes, there is also a significant reduction in forced vital capacity (FVC) and forced expiratory volume in one second (FEV1), and this impaired pulmonary function is significantly associated with raised plasma glucose concentration (Lange et al., 1989; Yeh et al., 2008). Finally, it is important to recognize that during influenza virus pandemics of the last century (including the 2009 H1N1 pandemic) the majority of fatalities were the result of secondary bacterial infections, rather than primary viral pneumonia (Short et al., 2012). Elevated airway glucose concentrations can increase the replication of respiratory bacterial pathogens (Garnett et al., 2013), suggesting that patients with diabetes may have increased bacterial outgrowth after an influenza virus infection. However, to the best of our knowledge, there have been no large-scale clinical studies investigating the association between influenza severity (and the acquisition or development of any associated secondary bacterial infection) and hyperglycemia in patients with diabetes. Thus, at present, the role of hyperglycemia in the pathogenesis of influenza virus remains unclear.

## A Role for Glycemic Oscillations

In the context of vascular complications of diabetes (e.g., cardiovascular disease), there is a growing body of evidence indicating that glucose variability is an important contributing factor to disease development (Hirakawa et al., 2014). In healthy individuals, blood glucose levels are kept within a narrow range of 4.4–6.7 mmol/L, including small and short-lived post-prandial peaks (Saisho, 2014). In the setting of impaired glucose tolerance, glucose fluctuations become greater and more frequent (Bonora and Muggeo, 2001). Blood glucose variability generally refers to hour-to-hour or day-to-day oscillations, but may also refer to month-to-month or even year-to-year changes. Glycemic variability is induced by many different factors including consuming a meal, changes in exercise, weight, medication, diet, and sleep patterns (Davies, 2004). As hyperglycemia is typically measured by a patient’s HbA1c, individuals with steady-state and oscillating glucose levels are generally not differentiated in the clinic, making prevalence estimates difficult to obtain. However, it is clear from clinical studies that the extent to which these glucose fluctuations occur differs greatly from patient to patient (Monnier et al., 2006).

There is now a growing body of evidence showing that glycemic oscillations play an important role in endothelial dysfunction, irrespective of HbA1c levels (Risso et al., 2001; Quagliaro et al., 2003; Azuma et al., 2006; Ceriello et al., 2008). For example, patients with type 2 diabetes that had blood glucose levels oscillating between 15 and 5 mmol/l every 6 h for 24 h had a significant increase in endothelial dysfunction relative to diabetic patients exposed to continuous 10 mmol/l glucose (Ceriello et al., 2008). *In vitro*, human umbilical endothelial cells (HUVECs) exposed to oscillating glucose have an increased level of apoptosis compared to HUVECs exposed to constant high levels of glucose (Quagliaro et al., 2003). Similarly, human endothelial cells exposed to intermittent high glucose had increased expression of ICAM-1, VCAM-1, VEGF, high mobility group box 1 (HMGB1), IL-8, NF-κB and E-selectin relative to endothelial cells exposed to stable high glucose (Quagliaro et al., 2005; Mudaliar et al., 2014). This is consistent with studies demonstrating increased monocyte adhesion to endothelial cells in rats exposed to glucose fluctuations relative to stable hyperglycemia (Azuma et al., 2006).

Endothelial cells, whilst not the primary target of influenza virus in humans, play an important role in disease pathogenesis (Teijaro et al., 2011; Short et al., 2013, 2014, 2016). During severe influenza virus infection, pulmonary endothelial cells produce cytokines which drive pulmonary lesions and mortality (Teijaro et al., 2011). In addition to mediating cytokine production, endothelial cells also indirectly control the inflammatory response in the lung during influenza virus infection via the expression of adhesion molecules (e.g., E-selectin, P-selectin, ICAM1, and VCAM1) (Short et al., 2014). Overexpression of these adhesion molecules is thought to impair pulmonary function during influenza virus infection by allowing the uncontrolled extravasation of leukocytes in the alveolus (Perrone et al., 2008; Short et al., 2014). These leukocytes can in turn damage the lung and impair respiratory function (Short et al., 2014). Given that glycemic oscillations are known to induce endothelial cytokine production (Quagliaro et al., 2005; Mudaliar et al., 2014) and enhance the expression of endothelial adhesions (Quagliaro et al., 2005; Mudaliar et al., 2014), it is tempting to speculate that glycemic variability augments the severity of influenza, at least in part, via effects on pulmonary endothelial cells.

## Conclusion and Future Directions

Diabetes is one of the world’s fastest growing chronic diseases, whereby the proportion of adults with diabetes is projected to increase from 9 to 10% by the year 2040 (International Diabetes Federation, 2015). With advancements in awareness, detection, and management of the disease, the average life expectancy of patients with diabetes is increasing (Lutgers et al., 2009; Guja et al., 2011; Huo et al., 2016). Thus, the number of individuals living with long-term complications is enhanced. Given this growing prevalence of diabetes and the increased window of opportunity for influenza virus infection, it is surprising that there are only few published studies in this field. It is therefore imperative that we dedicate research efforts to understanding how diabetes can increase the severity of influenza.

This includes delineating the role of other underlying comorbidities, hyperglycemia and glycemic oscillations in disease development and severity. Moreover, whilst type 1 and type 2 diabetes share a common symptom, i.e., hyperglycemia, they are vastly different in disease pathogenesis and, potentially, in their susceptibility to complications including influenza virus. Therefore, future studies would benefit from studying the development and severity of influenza in both type 1 and type 2 diabetes mellitus. This research will prove vital in mitigating the burden of future influenza epidemics and pandemics.

## Author Contributions

Conceived, wrote, and approved the manuscript: KH, LG, and KS.

## Conflict of Interest Statement

The authors declare that the research was conducted in the absence of any commercial or financial relationships that could be construed as a potential conflict of interest.
